# Ulnar nerve at the elbow – normative nerve conduction study

**DOI:** 10.1186/1749-7221-8-2

**Published:** 2013-02-11

**Authors:** Edvard Ehler, Petr Ridzoň, Pavel Urban, Radim Mazanec, Marie Nakládalová, Bohumír Procházka, Hana Matulová, Jan Latta, Pavel Otruba

**Affiliations:** 1 Department of Neurology, Regional Hospital and Faculty of Health Studies, University of Pardubice, 44 Kyjevská, 532 03, Pardubice, Czech Republic; 2 Department of Neurology, Thomayer University Hospital, Praha, Czech Republic; 3 Department of Occupational Medicine, 1st Faculty of Medicine, Charles University, and General University Hospital, Praha, Czech Republic; 4 National Institute of Public Health, Praha, Czech Republic; 5 Department of Neurology, 2nd Faculty of Medicine, Charles University, and University Hospital Motol, Praha, Czech Republic; 6 Department of Occupational Medicine, Faculty of Medicine and Dentistry, Palacký University, and University Hospital, Olomouc, Moravia; 7 Department of Neurology, Faculty of Medicine, Charles University, Hradec Králové, Czech Republic; 8 Department of Neurology, Faculty of Medicine and Dentistry, Palacký University, and University Hospital, Olomouc, Moravia

## Abstract

**Introduction:**

A goal of our work was to perform nerve conduction studies (NCSs) of the ulnar nerve focused on the nerve conduction across the elbow on a sufficiently large cohort of healthy subjects in order to generate reliable reference data.

**Methods:**

We examined the ulnar nerve in a position with the elbow flexion of 90^o^ from horizontal. Motor response was recorded from the abductor digiti minimi muscle (ADM) and the first dorsal interosseous muscle (FDI).

**Results:**

In our sample of 227 healthy volunteers we have examined 380 upper arms with the following results: amplitude (Amp)-CMAP(wrist) for ADM 9.6 ± 2.3 mV, MNCV at the forearm 60.4 ± 5.2 m/s, MNCV across the elbow 57.1 ± 5.9 m/s.

**Discussion:**

Our study showed that motor NCSs of the ulnar nerve above elbow (AE) and below elbow (BE) in a sufficiently large cohort using methodology recommended by AANEM gave results well comparable for registration from FDI and ADM.

## Introduction

Reliable reference data for parameters of electrophysiological examination of the ulnar nerve (UNE) are prerequisite for detection of abnormality in UNE lesions. Several authors published such data. However, because of different methodical approaches, the results varied considerably.

The technique of the UNE electrophysiological examination is associated with an array of methodical problems, which have been handled differently by individual authors. For research purposes, sophisticated technique evaluating many parameters may be used. On the other hand, indication for referral to surgery should be based on easily feasible and reproducible parameters [1-4].

Motor NCSs are considered to be more sensitive when recording from FDI than from ADM [5-8]. In the electrophysiological investigation, it is necessary to consider both conduction block (even without conduction velocity slowing) and primary axonal lesion (sporadically in chronic lesions without CV slowing) [7,9,10]. The position of the elbow during examination determines the actual length of the nerve and pressure of neighboring structures (ligaments) on fascicles and axons [5,11,12]. The elbow position in moderate flexion of 90° and in slight supination of the forearm guarantees constant tension of the nerve and constant compression by surrounding structures [5,11,13]. For the NCSs across elbow, many authors recommended the segment length of 10-cm as optimum [5]. The stimulating electrode should not be located at distance longer than 4 cm below the epicondyle. At longer distance, the nerve buries below FCU and the stimulation is no longer direct or the stimulus intensity is not supramaximal. Consequently, the amplitude of CMAP is lower [5,13-15]. Stimulation at 6 cm proximally from the medial epicondyle brings no substantial technical problems. Measurement on the segment of 10 cm length, recommended by most authors, is associated with the lowest error and is still able to detect focal conduction abnormality [11,15]. Due to specific arrangement of different fascicles in the UNE at the elbow, the conduction block of the fibers for FDI is more frequent than the block of the fibers for ADM. Likewise, the atrophy of FDI develops earlier than the atrophy of ADM in severe cases [16-19].

Whether the absolute value of CV across the elbow is more important for detection of UNE lesion at the elbow than the relative decrease of CV across the elbow in comparison with CV at the forearm, is a matter of discussion [5,11]. The relative diagnostic value/importance of the conduction block of fibers for ADM or FDI has not yet been established. It is questionable, what extent of the relative decrease in amplitude (20% or 25%?) should be regarded as a sign of partial conduction block and whether the temporal extension of CMAP should be taken into consideration [11]. For evaluation of sensory nerve conduction, the amplitude of SNAP recorded from digit V is especially important, while SNAP recorded from r. dorsalis or r. palmaris is of secondary significance. SNCV in the distal segment contributes only to the detection of a neuropathy or to the diagnosis of a lesion in the Guyon's canal. SNCV across the elbow is of no localizing value [7].

The prior NCSs on UNE conduction across the elbow addressed only some of the above-mentioned problems. Frequently, they did not meet criteria for standard examination, neither sufficient size of the studied group. Therefore, we decided to perform a study, which would try to eliminate the shortcomings. The goal of our study was to generate reliable normative data based on the examination of sufficiently large cohort of healthy subjects using standardized and reproducible technique for the UNE examination.

## Subjects and methods

### Subjects

The source study population included 227 disease free volunteers – 126 females and 101 males. All of them were of the Caucasoid race. Subjects ranged in age from 20 to 67 years with the average of 39 years. 214 subjects were right-handed and 13 were left-handed (Table 1). All participants provided written informed consent that had been previously approved by the institutional Ethical Committee.

**Table 1:** Demographic characteristics of the study group

	**Age (yrs)**		**Laterality**		**Total number of persons**	**Total number of evaluated hands**
**Gender**	**Range**	**Mean**	**Left-handed**	**Right-handed**		
**Males**	21-67	38,4	8	93	101	170
**Females**	20-66	40,2	5	121	126	210
**Total**	20-67	39,4	13	214	227	380

Among the primary exclusion criteria were medical conditions known as associated with polyneuropathy or mononeuropathy of the upper arms (diabetes mellitus and other metabolic disorders, malignancies, abuse of alcohol or other neurotoxic drugs, injuries of the nerves in the upper extremities), clinical signs of the ulnar or median nerves mononeuropathy, or cervical radiculopathies.

Subjects who passed the initial screening based on clinical history and physical examination then underwent the NCSs. The subjects with electrophysiological signs suggestive of a polyneuropathy were excluded. Also subjects with Martin-Gruber anastomosis were not considered eligible for the study, because of the influence of the anomaly on the CMAP amplitude.Eventually, 380 extremities entered into the normative database.

### Methods

Five EMG laboratories participated in the study. All NCSs were performed using EMG Medelec Synergy device and unified technique of examination. Surface stimulation electrodes were used. CMAPs were registered with single-use surface electrodes with the recording electrode placed above the motor point; SNAPs were registered using ring electrodes with conductive gel. Duration of stimulation impulses was 0.2 ms for motor NCSs and 0.1 ms for sensory NCSs. Bandwidth of the amplifier was set from 3 Hz to 10 kHz for MNCSs and from 20 Hz to 2 kHz for sensory NCSs.

The ulnar nerve was stimulated at the wrist, below the elbow, and above the elbow. Recording electrodes were placed above ADM and FDI. The median nerve was stimulated at the cubital fossa and at the wrist., the recording electrode was located above APB. The examination was performed in a position of the upper extremity with the elbow flexion of 90^o^ from horizontal throughout the whole measurement. To measure the distal motor latency of UNE, the distance between cathode of the stimulation electrode at the wrist and the active electrode above ADM was 8 cm, or 13 cm to the active electrode above FDI (measured along the course of UNE). To measure the distal motor latency of the NM, the distance between cathode of the stimulation electrode at the wrist and the active electrode above the belly of APB was 8 cm (measured along the course of NM). The stimulating electrode for MNCSs of the UNE below the elbow was located 4 cm distally from the center of the line connecting the olecranon and the medial epicondyle, in the direction to the ulnar styloid processus. The stimulating electrode for MNCSs of the UNE above the elbow was placed 6 cm proximally from the above-mentioned line, in the direction to the intramuscular sulcus. Sensory nerve conduction was measured antidromically, for UNE on digit V, with the distance between the stimulation and recording electrodes of 14 cm. For the NM it was measured on digit II, with the distance stimulation-recording 16-cm. Individual SNAPs were averaged. Latency was measured to the onset of the negative deflection, amplitude to its peak. Limb temperature was measured at the base of digit V and was maintained at a minimum of 32.0°C.

### Statistical analysis

The statistical package SPSS version 18 was used to perform the analyses.

For all neurophysiological parameters, descriptive statistics were calculated, namely median, arithmetic mean, standard error of the mean, and standard deviation. Kolmogorov-Smirnov goodness-to-fit test showed that the distribution of some parameters – mainly of amplitudes – significantly deviated from normal distribution. Therefore, the percentile method was used to set reference intervals. The lower and upper limits of the reference ranges were defined as 2.5^th^ and 97.5^th^ percentiles, respectively. Paired two-sample Student’s t-test was used to assess the statistical significance of the differences between corresponding parameters in both hands.

The level of the statistical significance was set at p≤0.05.

## Results

Statistical analysis included the following steps:

1. Calculation of the descriptive statistics for the parameters of motor conduction of the UNE recorded from ADM and FDI and for the NM recorded from APB

2. Calculation of the descriptive statistics for the parameters of sensory conduction of the UNE recorded from digit V and for NM recorded from digit II

3. Comparison of CMAP amplitudes in the right and left UNE recorded from ADM and FDI and comparison of CMAP amplitudes in the dominant and non-dominant hands Table 2, 3, 4, 5

**Table 2:** Shows descriptive statistics for the parameters of motor conduction of the UNE, recorded from ADM and from FDI

**Ulnar motor nerve conduction parameters**	**Recorded from ADM, dist. 8 cm**		**Recorded from FDI, dist. 13 cm**	
**Parameter**	**Mean [SD]**	**Reference range for 95% tolerance interval**	**Mean [SD]**	**Reference range for 95% tolerance interval**
DML wrist [ms]	2.8 [0.3]	2.2-3.4	3.4 [0.4]	2.4-4.3
MNCV forearm [m/s]	60.4 [5.2]	49.6-71.1	59.7 [4.7]	49.8-69.6
MNCV elbow [m/s] ADM	57.1 [5.9]	44.8-69.3	56.5 [5.7]	44.5-68.5
MNCV forearm – over the elbow [m/s]	3.3 [6.6]	−10,5-17,1	3,2 [6.4]	−10,2-16,5
Amp-CMAP wrist [mV]	9.6 [2.3]	4.9-14.4	12,0 [4.0]	3.5-20.4
Amp-CMAP BE [mV]	9.4 [2.1]	5.0-13.8	11.4 [3.9]	3.3-19.5
Amp-CMAP AE [mV]	9.2 [2.0]	5.1-13.3	11.4 [3.8]	3.5-19.3

Limits of reference range for each parameter were calculated as 2.5^th^ and 97.5^th^ percentile, respectively,. The lower limit of the reference interval for motor nerve conduction velocity (MNCV) of the UNE measured to ADM was 51.0 m/s at the forearm and 45.5 m/s across the elbow. The upper limit for the distal motor latency was 3.4 ms over the distance of 8 cm, the lower limits for CMAP recorded from ADM with stimulation points at the wrist, below the elbow (BE), and above the elbow (AE) were 5.6 mV, 5.8 mV, and 5.9 mV, respectively. The lower limit of that interval for MNCV measured to FDI at the forearm was 50.6 m/s, and 45.5 m/s across the elbow. The upper limit for the distal motor latency was 4.3 ms. The lower limits for CMAP recorded from FDI with stimulation points at the wrist, BE, and AE were 5.2, mV, 5.2 mV, and 5.6 mV, respectively.

**Table 3:** Shows descriptive statistics for two parameters of sensory conduction of the UNE, recorded from digit V, namely sensory nerve conduction velocity (SNCV) and amplitude of SNAP

	**Mean [SD]**	**Reference range (95% tolerance interval)**
SNCV [m/s]	55.8 [4.8]	45.9-65.8
Amp-SNAP [μV]	24.3 [11.6]	9.5-48.6

In the last column, reference ranges for each of the two parameters were defined with 2.5^th^ and 97.5^th^ percentile. The lower limit of the interval for SNCV was 47.9 m/s.

**Table 4:** Shows descriptive statistics for the parameters of motor and sensory conduction of the NM, recorded from APB for motor conduction and from digit II for sensory conduction

**Median nerve conduction parameters**	**Mean [SD]**	**Reference range (95% tolerance interval)**
DML wrist 8 cm [ms]	3.54 [0.34]	2.82-4.26
MNCV forearm [m/s]	57.1 [4.6]	47.6-66.7
A-CMAP wrist [mV]	10.0 [3.0]	3.7-16.6
A-CMAP elbow [mV]	9.7 [3.0]	3.4-16.0
SNCV [m/s]	55.8 [4.7]	45.9-65.6
Amp-SNAP [μV]	25,7 [4,11]	8.0.-51.7

In the last column, reference ranges defined with 2.5^th^ and 97.5^th^ percentile are given for each parameter.

**Table 5:** Compares MNCV across the elbow of the ulnar nerves in the dominant and non-dominant hands

**MNCV elbow [m/s]**	**Mean [SD]**	**Significance of the difference**
**Dominant** hand	57.5 [5.7]	p=0.042
**Nondominant** hand	56.8 [6.0]	

The mean MNCV at the dominant hands was by 0.69 m/s higher than the mean MNCV in the non-dominant hands. That minute difference was of a borderline statistical significance.

## Discussion

Several authors strove to compile normative data for motor and sensory NCSs in the ulnar nerve. The studies differed in the selection of healthy subjects, as well as in focusing on various electrophysiological parameters, and in using different methods. In addition, the number of the examined hands was mostly not very large [20,21].

Motor NCV of the UNE at the elbow segment is the principal parameter to be evaluated if suspicion on a lesion of the ulnar nerve at the elbow arises. It can be easily examined, providing that methodical principles are respected [5,22-24]. Acute UNE lesion in the elbow is of demyelinating type in about 60% of cases, with focal MNCV slowing across the elbow and partial conduction block [25,26]. There is also primary axonal lesion in about 40% of acute cases [27]. Chronic lesions caused by compression or corresponding to the cubital tunnel syndrome are usually marked by slowing of MNCV, frequently accompanied by decrease in amplitude and by temporal dispersion of CMAP [28,29]. It implies that MNCV across the elbow segment should be considered as a crucial parameter for the diagnosis of the lesion.

Conduction velocity across the elbow strongly depends on the position of the elbow (the angle of the flexion in the elbow). If the elbow is fully extended, the measured segment length is shorter than the true length of the nerve. Therefore, the measured CV is artifactually slower than it is in fact. Extreme flexion in the elbow results in the stretching of the nerve or even in its subluxation out of its groove. In our study, we chose the position of the upper extremity with the elbow flexion of 90° from horizontal. This is well feasible and in agreement with the recommendation by American Association of Neuromuscular and Electrodiagnostic Medicine and other authors [11,13].

Focal slowing of the CV in the across elbow segment can be expressed in two ways: (1) either as the absolute CV value to be compared with normal reference values for the across elbow segment or (2) as the relative (in percents) decrease in CV at the across elbow segment in comparison to the CV at the forearm. Because it has not yet been decided which of the two options is more sensitive, we recommend to evaluate both of them. Moreover, it should be kept in mind that the CV is influenced by many factors, such as age, anatomical relations in the region of the ulnar sulcus, and conduction velocity of other nerves in the particular person.

Benatar et al. (2009)[1] examined 100 healthy subjects and reported the following normative values for **conduction velocity** of the UNE **at the forearm**: A-CMAP for ADM 10.6 ± 2.5 mV, MNCV 60.8 ± 5.4 m/s, A-SNAP 29.8 ± 17.6 μV, SNCV 52.4±4.1 m/s. Buschbacher (1999)[30] found in 248 subjects A-CMAP for ADM 11.6 ± 2.1 mV, MNCV 61 ± 5 m/s. Oh, (1984) found in 40 subjects A-CMAP ADM 11.5 ± 2.5 mV, MNCV at the forearm 61.2 ± 5.3 m/s, A-SNAP 22.7 ± 14.4 uV, SNCV 60.9 ± 5.2 m/s.

In our cohort of 227 healthy volunteers, in which 380 hands were examined, we received the following normative values: Amp-CMAP(wrist) for ADM 9.6 ± 2.3 mV, MNCV at the forearm 60.4 ± 5.2 m/s, across the elbow 57.1 ± 5.9 m/s, Amp-SNAP for digit V 24.3 ± 11.6 μV, and SNCV 55.8 ± 4.8 m/s. The motor and sensory CV in our study did not differ significantly from the results by Benatar and Buschbacher [1,30]. We are in agreement also with Oh concerning NCSs at the forearm. However, the results across the elbow cannot be compared because Oh used different position of the elbow – full extension of 180°.

A very important parameter is **the nerve conduction velocity across the elbow.** On average, the difference of MNCV at the forearm and MNCV across the elbow was 3.3 ± 6.6 m/s or 3.2 ± 6.4 m/s when recording from ADM or FDI, respectively (Table 2). The limit value of the reference range for the MNCV slowing across the elbow in comparison with MNCV at the forearm was 17.1 m/s or 16.5 m/s for ADM or FDI, respectively. In the study by Kothari et al. (1998)[8] on a group of 20 healthy subjects (segment length 10-cm, flexion of elbow of 90°), the difference of MNCVs at the forearm and across the elbow was 9.3 ± 3.9 m/s, with limit value of the reference range for slowing across the elbow of 17.1 m/s. Mean MNCV across the elbow was 64.6 m/s and at the forearm 73.9 m/s (SD was not reported). Kitzinger (2005) [31] examined 25 upper extremities and found MNCV across the elbow 51.0 ± 8.4 m/s, slowing in comparison with the forearm was 9.4 ± 6.4 m/s. Mean amplitude of CMAP was 13.5 ± 3.8 mV. Buschbacher (1999)[30] did not observe any significant difference in MNCV across the elbow and at the forearm. Both MNCV were 61 m/s.

The **CMAP amplitude** is another important parameter. In healthy subjects, there is only an insignificant difference between the amplitude of CMAP elicited by stimulation above and below the elbow, provided that cases of MGA were excluded [32]. This holds for registration from ADM (9.4 mV AE, 9.2 mV BE), and FDI (11.4 mV AE, 11.4 mV BE) (Table 2). It implies that possible decrease of the amplitude can also be used as a sensitive parameter for detection of UNE lesion at the elbow region. In their groups of healthy subjects, Kimura, DeLisa and Buschbacher reported as mean values of relative decrease in CMAP amplitude AE and BE 2%, 6%, and 3%, respectively. Those values are well comparable with our results. Decreased amplitude of CMAP at the wrist is usually considered as a sign of an axonal lesion. By virtue of the size of our cohort, we could perform statistical comparison of CMAP amplitude in the right and left hands, and in the dominant and non-dominant hands. For registration from ADM, there was a statistically borderline difference (p=0.05) between the CMAP amplitude at the right and left hands and at the dominant and non-dominant hands (p=0.002). However, the differences were insignificant for registration from FDI (Table 6). This finding might be interpreted as a manifestation of more developed musculature in the dominant, more burdened extremity. However, this assumption is applicable only for ADM, not for FDI.

**Table 6:** Amplitudes of CMAP of the right and left UNE and also in the dominant and non-dominant hand, recorded from ADM and FDI, respectively, are compared

**Comparisons of the ulnar amplitude of CMAP (wrist stimulation)**		
Registration	Mean [SD]	Significance of the difference
Right ADM	9,4 [2.1]	p=0.05
Left ADM	9,1 [2.1]	
Right FDI	11,3 [3.8]	NS
Left FDI	11,3 [4.1]	
Dominant hand ADM	10,0 [2.2]	p=0.002
Non-dominant hand ADM	9.4 [2.3]	
Dominant hand FDI	11.8 [4.0]	NS
Non-dominant hand FDI	11.8 [4.3]	

As for FDI, the difference between the right and left UNE was not significant (NS). However, the difference reached borderline statistical significance in the case of ADM. The mean amplitude of CMAP registered from the right ADM was slightly higher than in the case of the left UNE. For ADM, the amplitude of CMAP of UNE in the dominant hand was significantly higher than in the non-dominant hand. The difference for FDI was non significant.

**Sensory neurography** of UNE is of importance for diagnosis of lesions in the region of Guyon’s canal. Its diagnostic significance for lesions at the elbow is just secondary – it can specify the severity of the damage based on the decrease in amplitude [5,11]. An array of authors have published reference values for SNCV. Thanks to the size of our cohort, we were able to analyze side differences. UNE SNAP amplitude was 24.2 ± 10.8 μV and 24.5 ± 12.5 μV in the dominant and non-dominant hands, respectively. The difference was not statistically significant (Table 7). Salerno (1996) [33] found similar insignificant difference between the UNE SNAP amplitude in the dominant (33.6 ± 16.2 μV) and non-dominant hands (35.7 ± 17.2 μV).

**Table 7:** Compares amplitudes of SNAP in the UNE recorded from digit V, in the dominant and non-dominant hands

**Amp-SNAP V [μV]**	**Mean [SD]**	**Significance of the difference**
**Dominant** hand	24.2 [10.8]	NS
**Non-dominant** hand	24.5 [12.5]	

In this aspect, there was no significant difference between the two hands.

## Significance – Conclusion

The authors report normative data for motor and sensory nerve conduction velocity studies The data were generated by examination of 380 hands in the cohort of 227 healthy subjects. The examination was performed in a sitting position, with elbow flexion of 90°. A 10-cm segment across the elbow was evaluated. Distal stimulation site was 4 cm from the medial epicondyle, proximal stimulation site was 6 cm from the medial epicondyle. Recording electrodes were placed over ADM and FDI. Based on the results, the reference values were compiled for parameters: MNCV at the forearm and across the elbow, difference between MNCV at the forearm and across the elbow, CMAP amplitude when recording from ADM and FDI, and SNCV in the ulnar and median nerves.

## Abbreviations

AANEM: American Association of Neuromuscular and Electrodiagnostic Medicine; ADM: Abductor digit minimi; AE: Above elbow; Amp: Amplitude; APB: Abductor pollicis brevis; BE: Below elbow; CMAP: Compound muscle action potential; CV: Conduction velocity; FCU: Flexor carpi ulnaris muscle; FDI: First dorsal interosseus muscle; MNCV: Motor nerve conduction velocity; NCSs: Nerve conduction studies; NM: Median nerve; NS: Not significant; SNAP: Sensory nerve action potential; SNCV: Sensory nerve conduction velocity; SD: Standard deviation; UNE: Ulnar nerve; W: Wrist.

## Competing interests

The authors declare, that they have no competing interests.

## Authors’ contributions

EhlerE - author, main contributor, acquisition of data, analysis and interpretation of data, drafting the manuscript, revising and final approval.

Ridzon P - author, substantial contribution, acquisition of data, analysis and interpretation of data, drafting the manuscript, revising content, final approval.

Urban P - author, substantial contribution, analysis and interpretation of data, drafting the manuscript, revising content. 

Mazanec R - author, substantial contribution, acquisition of data, analysis and interpretation of data, drafting the manuscript, revising content.

Nakladalová M - author, sustantial contribution, acquisition of data, analysis and interpretation of data, drafting the manuscript, revising content, final approval.

Procházka B - author, analysis and interpretation of data, statistitian.

Matulová H - author, substantial contribution, acquistion of data, analysis and interpretation of data.

Latta J - author, substantial contribution, acquisition of data, analysis and interpretation of data, drafting the manuscript, revising content.

Otruba P - author, substantial contribution, acquisition of data, analysis and interpretation of data.
